# The route to improve the effectiveness of negative PSAs^☆^

**DOI:** 10.1016/j.jbusres.2020.10.028

**Published:** 2021-02

**Authors:** Jingjing Ma, Zichuan Mo, David Gal

**Affiliations:** a National School of Development, Peking University, Beijing 100871, China; b International School of Business and Finance, Sun Yat-sen University, Zhuhai, Guangdong 519082, China; c University of Illinois at Chicago, 601 S. Morgan St., Chicago, IL 60607, United States

**Keywords:** Public service announcement (PSA), Mood, Identity threat, Self-affirmation

## Abstract

To encourage people to behave in a more socially responsible or healthy way, many public service announcements (PSAs) dramatically illustrate the dire consequences of undesirable social or health behaviors. However, although informative, such negatively-framed PSAs can generate unintended consequences (e.g., a bad mood, low message acceptance). In this research, we investigated two approaches to improve the effectiveness of negatively-framed PSAs: (1) mood elevation and (2) self-affirmation. Using three studies with large samples of consumers, we found that adding mood-elevating or self-affirming elements to a negative PSA can be an effective way to enhance message acceptance. This is especially the case for recipients currently engaging (vs. not engaging) in the undesirable behaviors. Additionally, we designed mood elevation methods that can be implemented in PSAs in practice.

## Highlights

• Negatively-framed PSAs can bring about an impaired mood and low message acceptance.
• Mood elevation and self-affirmation can increase the acceptance of negative PSAs.
• Self-affirmation can increase message acceptance without reducing identity threat.
• The two routes are more effective for people engaging (vs. not engaging) in the misbehaviors.

## 1 Introduction

Public service announcements (PSAs) are ubiquitous across media outlets, such as TV, magazines, social media, and billboards. In the US alone, approximately $1.8 billion worth of media are donated to PSAs each year. On average, each PSA campaign takes about nine months to develop and garners $25 million to $30 million in donated media (Ad Council). These PSAs usually focus on important social issues (e.g., smoking, drug abuse, drunk driving, online piracy). Such PSAs play an important role in communicating these issues to the public, in changing undesirable behaviors (e.g., reducing smoking among existing smokers), and in preventing the adoption of such behaviors in the future (e.g., reducing smoking adoption among non-smokers; Franke, 1994, Yang et al., 2019).

PSAs typically use either positive or negative message framing (Block and Keller, 1995, Maheswaran and Meyers-Levy, 1990). Negative PSAs illustrate the danger and dire consequences of misbehaviors. For example, a negative anti-smoking PSA might show how a person lost her teeth and jaw after years of smoking (which caused oral cancer), as well as the damage caused to her family, friends, job, and future (CDC, 2018). In contrast, positive PSAs emphasize the benefits of recommended behaviors. For example, the PSA message described above might be communicated by showing a desirable life in the absence of smoking. Many PSA producers adopt negative framing, aiming to create shocking visuals to illustrate dramatically the irresponsibility of viewers’ current behaviors and to encourage viral sharing via social media (Nichols, 2012). A natural question is: how effective are such negative PSAs?

On the one hand, because negative PSAs so vividly illustrate the dire consequences of misbehaviors, viewers engaging in such behaviors may experience cognitive dissonance and thus be motivated to change their behaviors (Festinger, 1962). On the other hand, as practitioners and researchers have discovered, cognitive dissonance can be resolved in more than one way. Dissonance and the identity threat induced by negative PSAs may instead encourage people to downplay their vulnerability to the danger, in turn, leading them to continue participating in the misbehaviors (e.g., Bensley and Wu, 2006, Gibbons et al., 1997). In fact, a *meta*-analysis showed that negative PSAs are, on average, less effective than positive ones in terms of message acceptance (O’Keefe & Jensen, 2008).

Despite the potential identity threat and dissonance they may produce, negative PSAs are sometimes necessary to communicate useful information to the public (e.g., the potential risks of certain behaviors). In addition, because negative information usually can engender more attention and induce stronger emotional, behavioral, and cognitive responses than positive information (Baumeister et al., 2001, Rozin and Royzman, 2001, Vaish et al., 2008), negative PSAs can usually benefit from increased dissemination through word-of-mouth and on social media and thus increase awareness (Stieglitz and Dang-Xuan, 2012, Stieglitz and Dang-Xuan, 2013). Owing to the necessity and benefits of negative PSAs, it is important for practitioners to learn how to improve their effectiveness in terms of message acceptance. Accordingly, in the current research, we investigated methods for enhancing the effectiveness of negative PSAs in realistic settings. Moreover, we explored the underlying mechanism and boundary conditions of those methods.

## 2 Theoretical background

### 2.1 Positive PSAs and negative PSAs

The relative effectiveness of negative and positive messages has been investigated extensively. Some research has found that negative messages are more persuasive than positive ones (e.g., Kalichman and Coley, 1995, Leshner and Cheng, 2009, Meyerowitz and Chaiken, 1987). For example, Meyerowitz and Chaiken (1987) found that, when advocating that young women perform a breast self-examination, a negative pamphlet had a greater impact than a positive one on their attitudes, intentions, and behaviors. However, other work showed contradictory evidence (e.g., Dooley et al., 2010, McCall and Ginis, 2004, Millar and Millar, 2000). For example, McCall and Ginis (2004) conducted a three-month follow-up study and found that patients receiving negative messages were less adherent to a cardiac rehabilitation exercise program and perceived more barriers to exercise than those receiving positive messages. A *meta*-analysis suggested that negative messages generally lead to less message engagement and lower persuasiveness than positive ones (O’Keefe & Jensen, 2008). In efforts to resolve these conflicting findings, moderators have been investigated. For example, research found that the relative effectiveness of the two types of PSAs differs depending on the regulatory focus of viewers (Teng, Zhao, Li, Liu, & Shen, 2019), level of elaboration in which individuals engage (Maheswaran and Meyers-Levy, 1990, Shiv et al., 1997), nature of consequences highlighted in the messages (Teng, Zhao, Wu, Fu, & Wang, 2019), and the kind of counterfactual thinking in which individuals engage (Baek, Shen, & Reid, 2013).

Although negative PSAs may sometimes be less effective than positive PSAs, they often contain more useful information than positive PSAs (Maheswaran and Meyers-Levy, 1990, Sherman et al., 2000). These negative messages are usually necessary for a PSA to fully convey its information. In addition, as mentioned earlier, negative PSAs, by providing shock value, might be more likely to capture one’s attention, raise awareness, and be shared than positive PSAs (Baumeister et al., 2001, Rozin and Royzman, 2001, Stieglitz and Dang-Xuan, 2012, Stieglitz and Dang-Xuan, 2013). Thus, an important issue is how to improve the effectiveness of negative PSA in terms of message acceptance. To address this issue, we first consider the reasons why negative PSA messages might be resisted.

Based on past research, we believe that there are two major factors that adversely impact the effectiveness of negative PSAs. First, negative PSAs might impair a recipient’s mood. Extant work has shown that people are less likely to accept counter-attitudinal messages and change their attitudes or behaviors when they experience a negative rather than a positive mood (Bless et al., 1990, Raghunathan and Trope, 2002). Because negative PSAs are likely to induce a bad mood, they may impede message acceptance. Second, scholars have observed that, when individuals perceive a message as a threat to their self-identity (i.e., an identity threat), they often respond defensively to the message (Liberman and Chaiken, 1992, Schüz et al., 2013, Sherman et al., 2000). Threatening PSA messages refer to those imperiling people’s positive self-image; they usually contain fearful, assertive, dogmatic, and self-relevant information (Janis and Feshbach, 1954, Liberman and Chaiken, 1992, Sherman et al., 2000). For example, Liberman and Chaiken (1992) found that people selectively criticize threatening health-related information--they are more critical and defensive toward highly threatening (e.g., highly assertive about the link between caffeine consumption and fibrocystic disease) parts than to less threatening (e.g., less assertive about the link) parts of the information. Because negative PSAs are likely to contain threatening messages (e.g., ominous consequences of smoking), they are likely to induce an identity threat. Therefore, people are likely to engage in defensive processing of negative PSAs. In sum, past research has revealed that an adverse mood and identity threat are two potential factors that reduce the effectiveness of negative PSAs in terms of message acceptance. Accordingly, taking these two factors into consideration, we propose two means for improving the effectiveness of negative PSAs.

### 2.2 Mood elevation and message acceptance

One alternative to increase acceptance of threatening negative PSAs is mood elevation. Negative or counter-attitudinal information is usually aversive. People need psychological resources to buffer or compensate for this aversiveness (Das, Vonkeman, & Hartmann, 2012). Past research showed that a positive mood is one resource that can buffer or compensate for the aversive consequences of negative information; it thus promotes attention to, processing of, and acceptance of such information (e.g., Bless et al., 1990, Das et al., 2012, Kaspar et al., 2015, Raghunathan and Trope, 2002, Roese and Olson, 2007, Tesser, 2000). For example, Das et al. (2012) found that smokers recalling positive events before viewing threatening health information were more likely to accept such information than those who previously recalled negative events. Kaspar, Gameiro, and König (2015) demonstrated that individuals in a negative mood were less willing to view negative information and less likely to remember such information than those in a positive mood. Similarly, Raghunathan and Trope (2002) showed that enhancing one’s mood enabled heavy caffeine consumers to elaborate more on negative but potentially relevant information about caffeine consumption, thus leading to a higher acceptance of this information. They surmised that “a positive mood provides the psychological buffer necessary to cope with self-relevant negative information—that is, when people are in a positive mood, they feel more confident of coping with the negative emotional impact of negative information” (Raghunathan & Trope, 2002, p. 511). Based on the above research, we propose:

**H1.** Adding a mood-elevating element to a negative PSA can increase the acceptance of the negative PSA by enhancing the recipients’ mood.

Much past research has examined the effect of mood elevation on message acceptance via an unrelated mood induction task—such as recall or essay writing (Das et al., 2012, Raghunathan and Trope, 2002). Although these methods can be easily implemented in lab settings, they are difficult to apply to PSAs in practice. One goal of our work is to design mood elevation methods that can be applied in actual settings to improve the effectiveness of negative PSAs.

### 2.3 Self-Affirmation and message acceptance

Another approach to increase acceptance of threatening negative PSAs is self-affirmation. Self-affirmation is the process of affirming or emphasizing positive aspects of one’s identity, often to offset one’s negative self-perceptions when one falls short of an ideal standard (Sherman & Cohen, 2006). Past research found that self-affirmation can increase the acceptance of negative or threatening messages by reducing or eliminating the perceived identity threat these messages cause (e.g., Cohen et al., 2000, Jacks and O’Brien, 2004, Sherman and Cohen, 2006, Tesser, 1988, Townsend and Sood, 2012). However, there are boundary conditions in which self-affirmation can reduce a perceived identity threat. For example, self-affirmation cannot decrease a perceived threat when the threatened domain is irrelevant or unimportant to the recipient (Boninger et al., 1995, Sherman et al., 2000), nor when the affirmed domain is not an important identity domain or is less important than the threatened domain for the recipient (Crocker et al., 2008, Koole et al., 1999, Sherman and Cohen, 2006). Because PSAs are usually made for the public, knowing and targeting each recipient’s important identity domain and offering different self-affirmations to different recipients is difficult for practitioners. Thus, the conditions identified by prior research for reducing an identity threat through self-affirmation may be challenging to achieve in practice.

If so, self-affirmation may not be able to reduce people’s identity threat induced by negative PSAs. However, we consider the issue of whether self-affirmation can increase message acceptance of negative PSAs even in the absence of a reduction in an identity threat. In the current research, we propose that self-affirmation can augment message acceptance by elevating mood, even when it is unsuccessful in reducing an identity threat. This proposition is based on past research suggesting that self-affirmation induces positive affect (Crocker et al., 2008, Koole et al., 1999, Tesser, 2000). For example, Koole et al. (1999) revealed that self-affirmation raised implicit positive affect, which in turn reduced the rumination engendered by an identity threat. Crocker et al. (2008) found that self-affirmation enhanced positive affect, especially other-directed affect (e.g., love, connectedness). Consistent with the “mood-as-a-resource” hypothesis, we expect that the reluctance to accept negative PSAs will be reduced by mood-elevating self-affirmation, even when the self-affirmation does not eliminate an identity threat. This suggests that mood elevation and threat reduction are two independent routes for self-affirmation to reduce one’s defensiveness to negative information. By identifying another route (i.e., mood elevation) through which self-affirmation can raise message acceptance, we provide PSA practitioners another means (i.e., mood-elevating self-affirmation) to improve message acceptance without the need to know recipients’ important identity domains. Thus, the following hypothesis is posited:

**H2.** Self-affirmation can increase the acceptance of negative PSAs by elevating the recipients’ mood.

### 2.4 Moderating role of target group

If a negative mood and an identity threat impair the effectiveness of negative PSAs, factors that strengthen/weaken the effect of negative PSAs on mood and identity threat should strengthen/weaken recipients’ reluctance to accept negative PSAs. We propose that the group targeted by a PSA (i.e., whether the recipient belongs to the group engaging in the undesirable behaviors identified in the PSA or not) is one such factor.

**Fig. 1:**
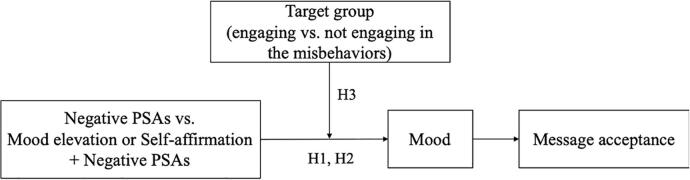
Conceptual model.

Although PSAs play an important role in changing and preventing undesirable social and health behaviors, they may have different effects depending on the recipient. For those engaging in the misbehavior (e.g., smokers), pointing out the adverse consequences of their current misbehaviors could threaten their self-identity or impair their mood. In contrast, for those currently not engaging in the misbehavior (e.g., non-smokers), the negative information is less likely to threaten their identity or impair their mood because they are not involved in “bad,” “irresponsible,” or “immoral” behaviors. Therefore, the effectiveness of negative PSAs may be lower for the former than for the latter group. This is consistent with extant work. For example, Sherman et al. (2000) found that, compared to non-coffee drinkers, coffee drinkers were more likely to reject information that stated the risk of caffeine on breast cancer. Furthermore, Bensley and Wu (2006) found that college students receiving a message recommending controlled drinking of alcohol tended to respond by increasing their drinking, particularly if they were heavy drinkers at the outset. Because mood elevation or self-affirmation should be more effective when the viewer’s mood or identity is threatened, we propose that inserting a mood-elevating or self-affirming element into a negative PSA would be more effective in improving message acceptance among the former than the latter group. Therefore, we postulate the following hypothesis (see Fig. 1 for the summarized conceptual model):

**H3**: The positive effect of inserting a mood elevation or self-affirmation element into negative PSAs is stronger for recipients engaging (vs. not engaging) in the identified undesirable behavior.

## 3 Study 1

The goal of study 1 was to test H1 and H2 by manipulating mood elevation and self-affirmation following exposure to a negative PSA. The negative PSA took the form of asking participants to reflect on their misbehavior.

### 3.1 Method

*Respondents.* Two hundred and sixty-two undergraduate students from a Midwestern university participated in this study in a behavioral laboratory.

*PSA manipulation.* The study focused on anti-piracy PSAs that are related to one’s moral identity. Respondents were randomly assigned to one of four PSA conditions: negative PSA, negative PSA + affirmation, negative PSA + mood elevation, and control condition. First, we manipulated negative PSAs by asking respondents about their past behaviors of using pirated content on the Internet. Those in the control condition did not answer these questions. Sample questions included: “Have you ever downloaded pirated software?”, “Have you ever downloaded pirated music?” (see Appendix A for all the questions). For each question, respondents chose either “yes,” “no,” or “choose not to answer”. Then, all the respondents were shown a message about pirating: “According to our prior research on piracy, many individuals believe that pirating is like stealing. It is unethical and irresponsible behavior that could lead to negative impacts on society. Do you agree with them?” It is not uncommon to see real-life PSAs asking people to reflect on their behaviors and then seeking to educate them by showing the dangers of those inimical behaviors. Previous research showed that identity threats can be caused by encouraging individuals to think about past behaviors that might be inconsistent with a positive identity standard (Boney-McCoy, Gibbons, & Gerrard, 1999) or emphasizing the aversive consequences of the discordance between behaviors and identity standards (Cooper & Fazio, 1984).

*Affirmation manipulation.* After the negative PSA manipulation, respondents in the negative PSA + affirmation condition were asked to write an essay to affirm their competence. Specifically, they were asked to “think of at least two achievements that reveal how competent and talented you are (e.g., receiving good grades, winning an award, or getting promoted at work),” and to “describe what you have achieved and how each accomplishment reflects your competence and success as a person.” The manipulation has been extensively used in past self-affirmation research (Cohen et al., 2000, Harris and Napper, 2005, Sherman et al., 2000, Shrira and Martin, 2005).

*Mood elevation manipulation.* After the negative PSA manipulation, respondents in the negative PSA + mood elevation condition were asked to write not about any achievements but about the experiences they might have on a sunny day: “Imagine that it has been raining for a week. When you wake up this Saturday morning, you see a beautiful sunny day. How happy do you feel? What do you plan to do on this sunny Saturday morning?” This task was intended to enhance respondents’ moods without providing any kind of identity affirmation.

*Mood measures.* All respondents then answered three questions concerning their mood (Huntsinger et al., 2009, Nakahara et al., 2009): “Overall, my mood now is___ 1 = very unpleasant 10 = very pleasant”; “How do you feel now? 1 = ☹ and 10 = ☺”; “How happy are you now? 1 = very unhappy 10 = very happy”.

*Perceived threat measure and message acceptance measure.* After the mood measurement, all respondents were asked to complete two tasks (order counterbalanced)—a brand choice task and a message acceptance task. The brand choice task was used as a measure of perceived threat. It was based on past research showing that threatened individuals were more likely to choose brands that symbolized the threatened domain (Cohen and Sherman, 2014, Rucker and Galinsky, 2008, Rustagi and Shrum, 2018). Respondents were asked to choose which of two brands (X or Y) they preferred. Brand X was described as being associated with competence (i.e., “in the business of victory, winning, and the feeling that comes with pushing the limits”); Brand Y was denoted as being associated with morality (i.e., “committed to reducing its environmental impact by using recyclable materials”).

Message acceptance was measured using two dimensions: attitude and behavioral tendency. Attitude was measured with two items using a 9-point scale (1 = strongly disagree, 9 = strongly agree): “Do you think piracy is an important issue?” “To what extent do you agree or disagree that pirating is like stealing?” Behavioral tendency was measured with two items using a 3-point scale: “To what extent do you think that you, personally, SHOULD NOT pirate?” “To what extent do you think that you, personally, will ACTUALLY NOT pirate?” (−1 = should not/will not, 0 = neutral, 1 = ok to pirate/will pirate; Sherman et al., 2000). Last, age, gender, education level, marital status, and income level were collected.

### 3.2 Results and discussion

Among respondents exposed to the piracy questions, 93.89 percent admitted to at least one of the piracy behaviors, suggesting that answering the piracy questions could threaten the moral identity of the bulk of respondents. As only 16 respondents did not engage in piracy behaviors, we were not able to investigate the moderating effect of target group. As such, we report only the main effects of PSA condition in this study.

*Message acceptance.* We first calculated an attitude index by averaging the two attitude items (*r* = 0.66, *p* < .01). We also calculated a behavioral tendency index (ranging from −2 to 2) by reverse-coding the two behavioral tendency items and summing them up (*r* = 0.58, *p* < .01). We conducted an ANCOVA with the four PSA conditions as an independent variable and the two dimensions of message acceptance (attitude and behavioral tendency) as dependent variables, respectively. Respondents’ gender, age, marital status, education level, and income level were added as covariates. For attitude, the results showed that gender had a significant effect on attitude, such that females had a more favorable attitude than males (5.35 vs. 4.81, *F*(1, 252) = 7.35; *p* < .01). More importantly, after controlling for the covariates, the effect of PSA condition on respondents’ attitudes toward anti-piracy statements was still significant (*F*(3, 252) = 6.72, *p* < .001). A pairwise comparison analysis demonstrated that those in the negative PSA + mood elevation condition reported a significantly more favorable attitude toward the anti-piracy statements than those in the negative PSA condition (5.54 vs. 4.37; *SE* = 0.28, *p* < .001). Similarly, respondents in the negative PSA + affirmation condition reported a significantly more favorable attitude than those in the negative PSA condition (5.36 vs. 4.37; *SE* = 0.29, *p* < .001). Also, respondents in the negative PSA condition reported a significantly less favorable attitude than those in the control condition (4.37 vs. 5.22; *SE* = 0.28, *p* < .01).

For behavioral tendency, the results were similar to those for attitude. Specifically, gender had a significant effect on behavioral tendency, such that males had a higher behavioral tendency than females (−0.01 vs. −0.32; *F*(1, 252) = 3.98; *p* < .05). More importantly, after controlling for the covariates, the effect of PSA condition on respondents’ anti-piracy behavioral tendency was still significant (*F*(3, 252) = 6.64, *p* < .001). A pairwise comparison demonstrated that those in the negative PSA + mood elevation condition reported a significantly higher behavioral tendency than those in the negative PSA condition (0.10 vs. −0.78; *SE* = 0.22, *p* < .001). Similarly, respondents in the negative PSA + affirmation condition reported a significantly higher behavioral tendency than those in the negative PSA condition (0.02 vs. −0.78; *SE* = 0.22, *p* < .001). Also, respondents in the negative PSA condition reported a significantly lower behavioral tendency than those in the control condition (−0.78 vs. −0.11; *SE* = 0.22, *p* < .01). These results suggest that both mood elevation and self-affirmation can increase the message acceptance of negative PSAs, thus lending support to H1 and part of H2 (see Fig. 2a, Fig. 2b).

**Fig. 2a:**
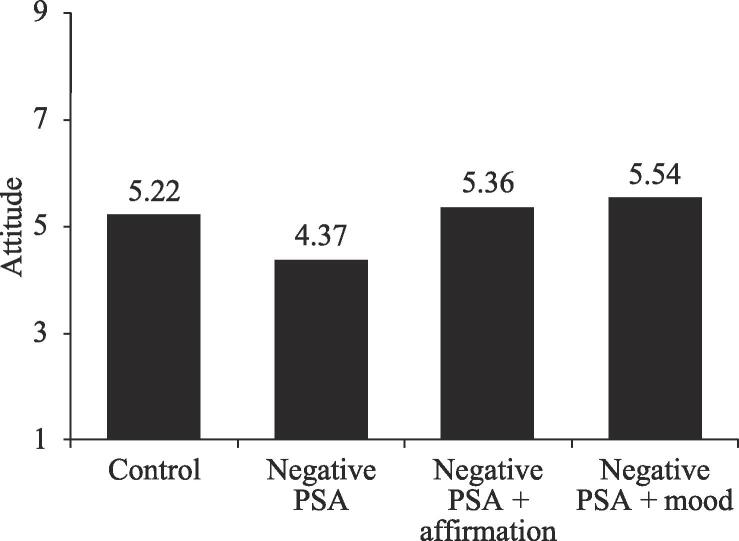
Message acceptance -- attitude, Study 1.

**Fig. 2b:**
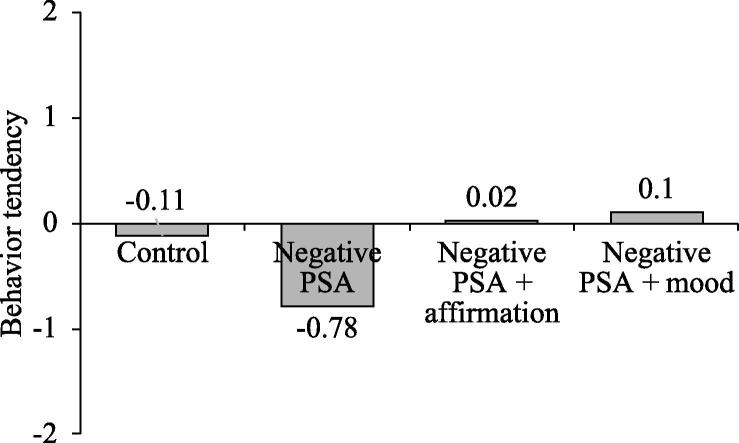
Message acceptance – behavioral tendency, Study 1.

**Fig. 3:**
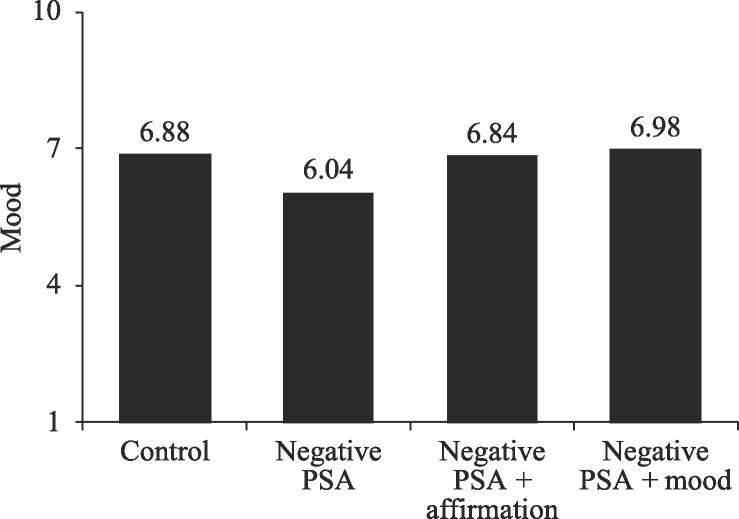
Mood, Study 1.

*Mood.* We analyzed respondents’ mood ratings by averaging the three mood measures (*Cronbach’s α* = 0.96). An ANOVA showed that respondents’ mood was significantly different across the four conditions (*F*(3, 258) = 4.27, *p* < .01). A pairwise comparison analysis showed a similar pattern to the pattern for message acceptance (see Fig. 3). Specifically, respondents in the negative PSA + mood elevation condition reported a significantly better mood than those in the negative PSA condition (6.98 vs. 6.04; *SE* = 0.30, *p* = .01); those in the negative PSA + affirmation condition also reported a significantly better mood than those in the negative PSA condition (6.84 vs. 6.04; *SE* = 0.30, *p* = .046). In addition, the mood in the negative PSA condition was lower than in the control condition (6.04 vs. 6.88; *SE* = 0.30, *p* = .03). Furthermore, mood ratings of those in the negative PSA + affirmation, negative PSA + mood elevation conditions, and control conditions did not differ significantly (*p*’s = 1.00). These results suggest that both self-affirmation and mood elevation enhanced respondents’ mood to the same level as those not viewing the negative PSA.

**Fig. 4:**
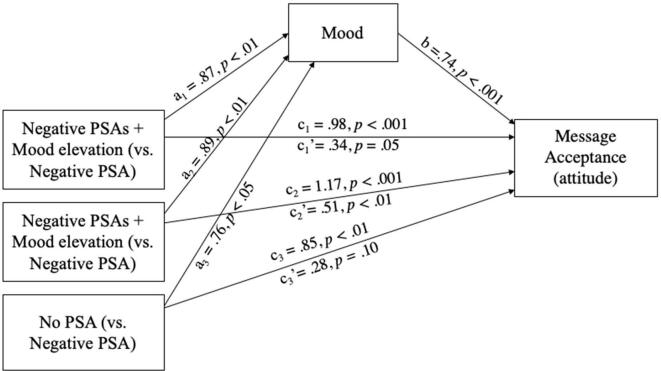
Mediation analysis, Study 1.

To further test the role of mood, we conducted a mediation analysis (PROCESS, model 4; Hayes, 2018) with PSA condition as an independent variable (dummy coded with the negative PSA condition as a baseline condition), mood as a mediator, attitude as a dependent variable, respondents’ gender, age, marital status, education level, and income level as covariates (only gender had a significant effect on attitude as reported in the main effect results). After controlling for these control variables, the results showed that relative to respondents in the negative PSA condition, those in the negative PSA + mood elevation condition (*a_1_* = 0.87, *p* < .01), negative PSA + self-affirmation condition (*a_2_* = 0.89, *p* < .01), and control condition (*a_3_* = 0.76, *p* < .05) all reported a better mood. Mood, in turn, was positively related with attitude (*b* = 0.74, *p* < .001). The relative indirect effects were all significant for the three conditions (*a_1_b* = 0.64, 95% CI [0.1991 to 1.0662]; *a_2_b* = 0.66, 95% CI [0.2373 to 1.0549]; *a_3_b* = 0.56, 95% CI [0.0887 to 1.0271]). In addition, the relative direct effects of mood elevation + negative PSA (*c_1_′* = 0.34, *p* = .05) and self-affirmation + negative PSA (*c_2_′* = 0.51, *p* < .01) on attitude were also significant (see Fig. 4). These results suggest that, compared to the negative PSA condition, both mood elevation and self-affirmation increased the attitude toward anti-piracy statements by enhancing mood, supporting H1 and H2. Attitude in the control condition was also higher owing to a better mood.

Similarly, we conducted another bootstrap analysis using the behavioral tendency measure as a dependent variable. Among all the control variables, only gender had a significant effect on behavioral tendency as reported in the main effect results. After controlling for the control variables, the results showed that relative to respondents in the negative PSA condition, those in the negative PSA + mood elevation condition (*a_1_* = 0.89, *p* < .01), negative PSA + self-affirmation condition (*a_2_* = 0.87, *p* < .01), and control condition (*a_3_* = 0.76, *p* = .01) all reported a better mood. Mood, in turn, influenced behavioral tendency (*b* = −0.21, *p* < .001). The relative indirect effects on behavioral tendency were all significant for three conditions (*a_1_b* = −0.19, 95% CI [−0.3385 to −0.0630]; *a_2_b* = −0.18, 95% CI [−0.3406 to −0.0524]; *a_3_b* = −0.16, 95% CI [−0.3266 to −0.0245]). In addition, the relative direct effects of the three conditions on behavioral tendency were also significant (*c_1_′* = 1.06, *p* < .001; *c_2_′* = 0.98, *p* < .001; *c_3_′* = 0.83, *p* < .001). In sum, the findings suggested that both mood elevation and self-affirmation increased message acceptance by enhancing mood, thus supporting H1 and H2.

**Fig. 5:**
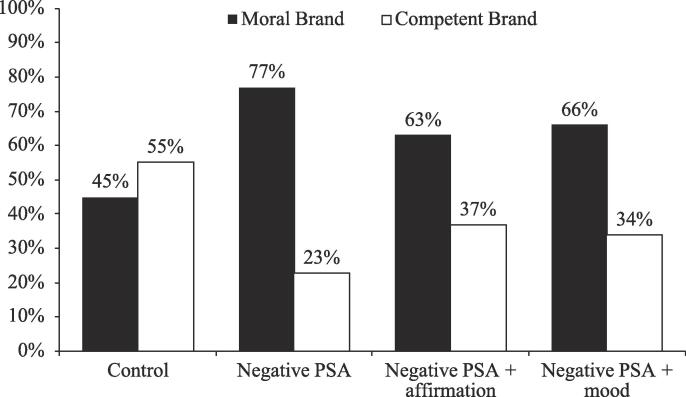
Perceived threat: Choice of moral brand, Study 1.

*Perceived threat.* Finally, we tested whether the increased message acceptance was also driven by a reduced identity threat. Chi-square analyses showed that the choice likelihoods for the moral brand were significantly different across the four conditions (χ^2^(3) = 14.76, *p* < .01). As shown in Fig. 5, relative to the respondents in the control condition, those in the negative PSA condition were more likely to choose the moral brand than those in the control condition (77% vs. 45%; χ^2^(1) = 14.09, *p* < .001). This suggested that a threat to moral identity was caused by the negative PSA (and thus a higher preference for the moral brand). Furthermore, respondents in the negative PSA + affirmation condition were also more likely to choose the moral brand than those in the control condition (63% vs. 45%; χ^2^(1) = 4.10, *p* = .04); plus, they did not significantly differ from respondents in the negative PSA condition (63% vs. 77%; χ^2^(1) = 3.16, *p* = .08). The same pattern appeared in the PSA + mood elevation condition: respondents in the negative PSA + mood elevation condition were more likely to choose the moral brand than those in the control condition (66% vs. 45%; χ^2^(1) = 5.69, *p* = .02), and they did not significantly differ from respondents in the negative PSA condition (66% vs. 77%; χ^2^(1) = 2.00, *p* = .16) nor in the negative PSA + affirmation condition (66% vs. 63%; χ^2^(1) = 0.14, *p* = .71). These results suggested that neither the self-affirmation nor the mood elevation manipulation mitigated the perceived identity threat in the morality domain (see also Rustagi & Shrum, 2018).

*Discussion*. Study 1 showed that both mood elevation and self-affirmation can increase the acceptance of a negative PSA. These effects can be explained by improved mood. In this study, self-affirmation augmented message acceptance by elevating mood, not by decreasing identity threat. This is likely because self-affirmation is only effective in reducing identity threat when the affirmed domain is important to recipients (Crocker et al., 2008, Koole et al., 1999, Sherman and Cohen, 2006). As mentioned before, PSAs are for the public, and important identity domains differ across individuals. Therefore, designing a single PSA to target everyone’s important identity domains is generally impossible. In contrast, we argue that mood-elevating self-affirmation can be useful for practitioners because it can increase PSA message acceptance by elevating mood, even when it fails to reduce identity threat. The results also revealed significant direct effects of mood elevation and self-affirmation on acceptance of the negative PSA, suggesting that there may be other factors driving the effect in addition to mood (see General Discussion for more on this issue).

## 4 Study 2

In study 2, instead of manipulating mood through independent mood induction tasks, we directly embedded the mood-elevating materials into PSAs in a way that can be easily implemented in practice. This study had two goals. One was to design a mood-elevating technique that can be directly implemented in practice to improve the effectiveness of negative PSAs. The second was to test H3. The study used a 2 (smoker vs. non-smoker) × 2 (negative PSA vs. negative PSA + mood elevation) between-subjects design.

### 4.1 Method

*Respondents.* Four hundred and forty-nine male adults were recruited through a nationwide online subject pool by a leading marketing research company in China. This subject pool contains>3.9 million registered members.

*Method.* This study focused on anti-smoking PSAs. First, we asked respondents about their smoking habits (whether they smoked and how frequently) and their knowledge about the possible consequences of smoking. Then, they were randomly assigned to one of two PSA conditions: a negative PSA condition or a negative PSA + mood elevation condition. In the negative PSA condition, respondents saw a negative PSA with a picture of a damaged lung and text describing the deadly consequences of smoking. After viewing the negative PSA, those in the negative PSA + mood elevation condition saw a poster with pictures of a happy family and beautiful natural scenery and with accompanying text affirming family values (e.g., “No matter what happens, the smile of families can always cheer us up”; see Appendix B for the stimuli). This manipulation was intended to elevate mood directly and affirm family values concurrently (see the 4.2 Results and Discussion part for a posttest of the manipulation stimuli). It was also easy to apply in practice.

Message acceptance was measured with five items tapping anti-smoking message attitude (3 items) and behavioral tendency (2 items; Block and Keller, 1995, Maheswaran and Meyers-Levy, 1990): “Smoking is indeed bad for health,” “Smoking can cause lung cancer,” “It is irresponsible to pass second-hand smoke to family members,” “I should quit smoking,” and “I will try to quit smoking as soon as possible” (1 = strongly disagree, 7 = strongly agree; *Cronbach’s α* = 0.76). We averaged the five items into one message acceptance index. Then, using a seven-point scale, we asked participants about the importance of valuing family (Schmeichel & Martens, 2005). Last, gender and age information was collected (all males; M_age_ = 33.4 years old).

### 4.2 Results and discussion

**Fig. 6:**
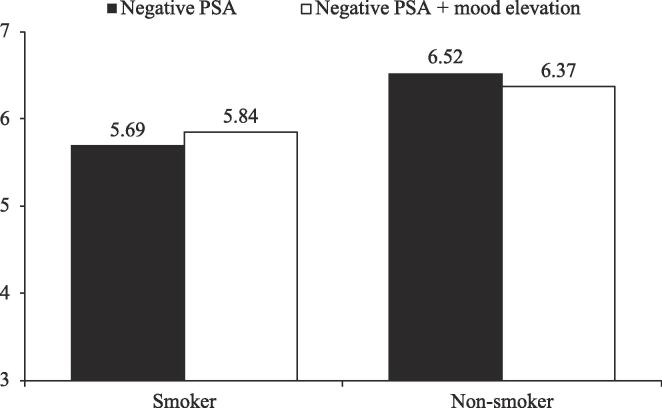
Message acceptance, Study 2.

*Message acceptance.* Seventy-three percent of the sample smoked. A two-way ANCOVA was conducted with the target group (smoker vs. non-smoker) and the PSA condition (negative PSA vs. negative PSA + mood elevation) as independent variables and message acceptance as the dependent variable. Respondents’ age, prior knowledge about the consequence of smoking, and the importance of family were added as covariates. The results showed a significant effect of respondents’ prior knowledge (*F*(1, 442) = 37.98, *p* < .001) and the importance of family value (*F*(1, 442) = 78.44, *p* < .001) on message acceptance. After controlling for these variables, the main effect of target group was also significant: non-smokers reported a higher message acceptance than smokers (6.45 vs. 5.77; *F*(1, 442) = 91.14, *p* < .001). More importantly, the results showed a significant interaction between the target group and PSA condition (*F*(1, 442) = 4.39, *p* = .04; see Fig. 6). Contrast analysis demonstrated that, for smokers, the mood elevation technique significantly increased message acceptance of the negative PSA (5.84 vs. 5.69; *F*(1, 442) = 4.01, *p* < .05); in contrast, for non-smokers, the message acceptance essentially did not differ between the two PSA conditions (6.37 vs. 6.52; *F*(1, 442) = 1.50, *p* = .22). These results supported H3.

*Stimuli Posttest*. A posttest was conducted to ensure that the negative PSA used in study 2 was indeed negative and threatening to smokers and that the mood elevation poster was mood-elevating and self-affirming, as intended. The test was undertaken using the same subject pool as in study 2 (N = 2106; all males; *M_age_* = 38.06, SD = 14.42). The results showed that respondents believed that the negative PSA emphasized the negative consequences of smoking rather than the benefits of quitting smoking (1 = totally emphasized negative consequences, 7 = totally emphasized benefits; *M* = 2.35 < 4, *t*(2105) = 43.28, *p* < .001). Additionally, respondents rated the negative PSA as unpleasant (1 = unpleasant, 7 = pleasant; *M* = 2.83 < 4, *t*(2105) = 33.80, *p* < .001), negative (1 = negative, 7 = positive; *M* = 3.03 < 4, *t*(2105) = 26.65, *p* < .001), and threatening to smokers (1 = very much, 7 = not at all; *M* = 2.63 < 4, *t*(2105) = 42.91, *p* < .001). Conversely, the mood elevation poster was rated as pleasant (1 = unpleasant, 7 = pleasant; *M* = 6.08 > 4, *t*(2105) = 87.14, *p* < .001), positive (1 = negative, 7 = positive; *M* = 6.11 > 4, *t*(2105) = 96.13, *p* < .001), and affirming of respondents’ family values (averaged two measures: “This poster reminds people of the warmth of family,” “This poster make people realize the value of family”; 1 = totally disagree, 7 = totally agree; *Cronbach’s α* = 0.74; *M* = 6.07 > 4, *t*(2105) = 100.97, *p* < .001).

*Discussion*. Study 2 found that inserting a mood-elevating self-affirmation into a negative anti-smoking PSA was more likely to increase message acceptance among smokers than among non-smokers. This is consistent with the proposed moderating role of the target group in H3. In addition, the study design incorporated a mood elevation manipulation in the form of pictures that can easily be applied in PSA practice.

## 5 Study 3

To increase the external validity of this research, study 3, using a contemporary globally important and currently relevant domain with a nationally representative sample, sought to test our main hypotheses: the main effect of mood elevation on the effectiveness of negative PSAs (H1), as well as the moderating role of target group (H3). Specifically, we tested PSAs intended to persuade Chinese consumers not to eat wild animals to prevent viruses passing from animals to humans (e.g., the Coronavirus).

Moreover, although mood-elevating elements of an intervention have sometimes been presented to participants following negative messages to compensate for the threat (e.g., Adams et al., 2011, Roese and Olson, 2007), another way past research has induced mood is by manipulating mood before the introduction of negative or threatening messages (e.g., Das et al., 2012, Raghunathan and Trope, 2002, Trope and Neter, 1994). Actually, the “mood-as-a-resource hypothesis” generally assumes that the positive mood is activated in advance to buffer future negative self-relevant information, and that individuals’ initial positive mood will be undermined after they process such negative information (Raghunathan & Trope, 2002). Therefore, another goal of study 3 was to figure out whether the positive effect of mood elevation remained if the mood elevation occurred before the negative information in the PSA was shown. We believe that this alternative offers a more conservative test of the mood elevation hypothesis as the mood elevation manipulation comes before the negative PSA. To elaborate, the manipulation starts with positive information but ends with negative information; this is a conservative test because the negative information and the attendant sense of threat might be more salient in mind when respondents are providing their message evaluation. Indeed, even if effective, we might expect the effectiveness of the mood manipulation to be weaker than when the manipulation ends with a positive tone as was the case in the previous studies.

### 5.1 Method

*Respondents*. To access a nationally representative sample, we collaborated with the leading market research company in China, InsightWorks (same as in study 2). InsightWorks has built a survey panel exceeding 3.9 million respondents from 31 provincial divisions of mainland China. We drew a national representative sample of 13,329 respondents from this pool, based on their gender, age, and location. As such, we drew a sample with a similar profile as the population of mainland China (see Table 1 for a comparison of the profile of the sample and the population in mainland China). Nine hundred and eighty-seven respondents did not complete the survey or pass the attention check question. The final sample size for analysis was 12,342 (48.7% female; *M_age_* = 37.85, SD = 14.40).

**Table 1:** Sample structure, Study 3.

Profile		Population (mainland China)	Full sample	Analysis sample
**Location** (area of mainland China)	Northern	20.51%	20.93%	18.8%
	Eastern	29.41%	29.40%	30.2%
	Western	21.82%	21.32%	20.3%
	Middle	16.13%	16.70%	16.1%
	Southern	12.13%	11.57%	14.5%
	Other (Hongkong, Macau, Taiwan, or other areas)		0.08%	
Sum		100%	100%	100%
**Gender**	Male	51.22%	51%	56.8%
	Female	48.78%	49%	43.2%
Sum		100%	100%	100%
**Age group** (years old)	< 25	33.66%	30.72%	26.2%
	25–34	14.87%	15.55%	16.4%
	35–44	18.21%	19.05%	21.5%
	45–54	13.83%	13.88%	13.3%
	>54	19.43%	20.8%	22.6%
Sum		100%	100%	100%

*Note.* Population data of the mainland China are from the census of National Bureau of Statistics.

*Method.* The study used a one-factor (PSA condition: negative PSA vs. mood elevation + negative PSA vs. no PSA control) between-subjects design. Respondents were randomly assigned to one of three conditions. In the negative PSA condition, they saw a negative PSA with pictures of wild animals, Coronavirus, and messages about the danger of eating wild animals (see Appendix C for the stimuli). In the mood elevation + negative PSA condition, we followed the procedure used in the studies of Das et al., 2012, Raghunathan and Trope, 2002 research; respondents went through a mood elevation manipulation before receiving negative or threatening information. They first saw pictures of the beautiful natural scenery of the spring season and positive messages (e.g., “The spring is coming, and the flowers are blooming; After the Corona epidemic, let us embrace the spring together”). This manipulation was intended to elevate mood and was adopted from real advertisements on TV and social media; those advertisements sought to convey positive information to the public about the Corona pandemic. After the mood elevation manipulation, respondents read the following instructions: “Although the pandemic is almost under control, we still need to remember the following lessons.” Then they viewed the negative PSA. We also recorded the time duration respondents spent watching the negative PSA; it did not differ between the negative PSA condition and the mood elevation + negative PSA condition (21.20 s vs. 19.54 s; *t*(8252) = 1.01, *p* > .1). In the control condition, respondents were not exposed to any PSAs. Then, all respondents rated their mood (1 = very unpleasant, 7 = very pleasant) as a manipulation check for the mood elevation manipulation.

We measured three sets of dependent variables: attitude, behavioral tendency, and voting behavior. Attitude was measured using three items on a 7-point scale (1 = totally disagree, 7 = totally agree): “Eating wild animals will cause the spread of viruses”; “Eating wild animals is irresponsible for the health of yourself and others”; “People should stop eating wild animals.” Behavioral tendency was measured with two items using a 7-point scale: “Will you eat wild animals in the future?”; “Will you stop others (such as your families or friends) from eating wild animals in the future?” Last, respondents were asked to vote on a “Ban on Eating Wild Animals” law in China (which a local government was soliciting on the Internet during the period of this study). There were five specific types of wild animals identified in the law: “Wild animals protected by past laws”; “Terrestrial wild animals that grow naturally in the wild”; “Terrestrial wild animals (e.g., turtles, rabbits, birds, insects) that are artificially bred and raised“; “Non-edible animals and animal-made products used for scientific research and experiments”; and “Frogs (e.g., bullfrogs).” Respondents voted on each using a 3-point scale (1 = oppose, 2 = neutral, 3 = support).

We also collected demographic information and asked respondents to self-report whether and how frequently they had eaten wild animals in the past (including birds, rabbits, boars, etc.; 1 = never, 4 = once a year, 7 = >10 times a year). Nearly 82% reported that they had never eaten any wild animals in the past, and 94.54% indicated a frequency of once a year or less.

### 5.2 Results and discussion

*Stimuli Pretest.* We conducted a pretest to ensure that the negative PSA used in study 3 was indeed negative and threatening to those eating wild animals and that the mood elevation manipulation stimulus was pleasant and positive (N = 133; 60.2% female; *M_age_* = 24.67, SD = 4.08). The results showed that respondents rated the negative PSA in this study as emphasizing the negative consequences of eating wild animals rather than the benefits of not eating wild animals (1 = totally emphasized negative consequences, 7 = totally emphasized benefits; *M* = 1.80 < 4, *t*(132) = 19.44, *p* < .001). Additionally, they rated the negative PSA as unpleasant (1 = unpleasant, 7 = pleasant; *M* = 2.29 < 4, *t*(132) = 16.78, *p* < .001), negative (1 = negative, 7 = positive; *M* = 2.52 < 4, *t*(132) = 13.44, *p* < .001), and threatening to people who like to eat wild animals (1 = very much, 7 = not at all; *M* = 2.00 < 4, *t*(132) = 17.32, *p* < .001). Conversely, respondents rated the mood-elevating stimuli as pleasant (1 = unpleasant, 7 = pleasant; *M* = 6.32 > 4, *t*(132) = 35.98, *p* < .001) and positive (1 = negative, 7 = positive; *M* = 6.26 > 4, *t*(132) = 32.38, *p* < .001).

*Sample for Analysis.* One goal of this study was to test the moderating role of target group (H3). Since only 5.46% of the full sample ate wild animals more than once a year, the sample size was severely unbalanced between the two target groups. Therefore, we used stratified sampling to randomly draw subsamples from the respondents not eating wild animals. Specifically, we kept respondents eating wild animals more than once a year as a target group (N = 674, 5.46% of all respondents). Then, we randomly drew a sample of 674 from the respondents not eating wild animals based on the sample size in each PSA condition of the target group (N = 223, 235, 216 in the control, negative PSA, and mood elevation + negative PSA condition, respectively) as the non-target group. We analyzed this subsample (N = 1348) and report the results of it here. In the Web Appendix, we report the results of the full sample (main effects), as well as the results of another subsample using a different stratified sampling method as a robustness check of the moderation effect results.

*Manipulation Check.* A one-way ANOVA showed that the three conditions resulted in significant differences in mood (*F*(2, 1345) = 55.69, *p* < .001). Specifically, those in the mood elevation + negative PSA condition reported a better mood than those in the negative PSA condition (4.16 vs. 3.69; *p* < .001), suggesting a successful mood elevation manipulation. Moreover, after viewing the negative PSA, respondents in both PSA conditions reported worse moods than those in the control condition (*M_control_* = 4.75; *p’s* < 0.001).

*Message Acceptance.* We first averaged the attitude and behavioral tendency items into two indices, respectively: attitude (3 items; *Cronbach’s α* = 0.85), behavioral tendency (2 items; *r* = 0.54, p < .01). For voting behavior, we recoded the responses to the 5 items into −1 (oppose), 0 (neutral), 1 (support). Then, we summed up the responses of the 5 items for each individual to form a voting behavior index, which ranged from −5 (opposes the law for all animals) to + 5 (supports the law for all animals). Next, we conducted a MANCOVA with PSA condition as an independent variable, the three indices as dependent variables, and age, gender, education, province (from Hubei Province or not), living area (urban or rural), number of children, and family income as covariates. The results showed that the effects of the control variables were all significant (*p*’s < 0.05) except for the living area (*p* > .1). Furthermore, after controlling for these variables, the main effect of the PSA condition (*F* = 2.22, Wilks’ Lambda = 0.99; *p* < .05) and target group (*F* = 147.40, Wilks’ Lambda = 0.75; *p* < .001) were both significant. Specifically, PSA condition had significant main effects on attitude (*F*(2, 1335) = 3.76, *p* < .05) and behavioral tendency (*F*(2, 1335) = 5.43, *p* < .01), but not on voting behavior (*F*(2, 1335) = 0.55, *p* > .1). The main effects of target group were significant on all three dependent variables (attitude: *F*(1, 1335) = 279.59, *p* < .001; behavioral tendency: *F*(1, 1335) = 385.95, *p* < .001; voting behavior: *F*(1, 1335) = 56.13, *p* < .001). Pairwise analyses on PSA condition showed that, for attitude, respondents’ attitude in the mood elevation + negative PSA condition was significantly more favorable than that in the control condition (5.88 vs. 5.67; *p* < .01); but neither of the attitudes in these two conditions differ from the attitude in the negative PSA condition (5.77; *p’*s > 0.1). For behavioral tendency, respondents’ behavioral tendency in the mood elevation + negative PSA condition was higher than that in the negative PSA condition (5.90 vs. 5.82, *p* < .05); and both PSA conditions induced a higher behavioral tendency than in the control condition (5.65, *p*’s < 0.05).

More importantly, the interaction between the PSA condition and target group was marginally significant (*F* = 1.88, Wilks’ Lambda = 0.99; *p* = .08), suggesting that the effect of PSA condition on the dependent variables differed between the target and non-target groups. Moreover, the findings revealed that the interaction was marginally significant on attitude (*F*(2, 1335) = 2.61, *p* = .07) and behavioral tendency (*F*(2, 1335) = 2.94, *p* = .05) but was non-significant on voting behavior (*F*(2, 1335) = 0.46, *p* > .1). Contrast analysis showed that the effect of the PSA condition on attitude was significant only for the target group (*F*(2, 1335) = 6.33, *p* < .01) but not for the non-target group (*F*(2, 1335) = 0.05, *p* = .95). Similarly, the effect of the PSA condition on behavioral tendency was significant only for the target group (*F*(2, 1335) = 8.07, *p* < .001) but not for the non-target group (*F*(2, 1335) = 0.31, *p* = .74).

**Fig. 7:**
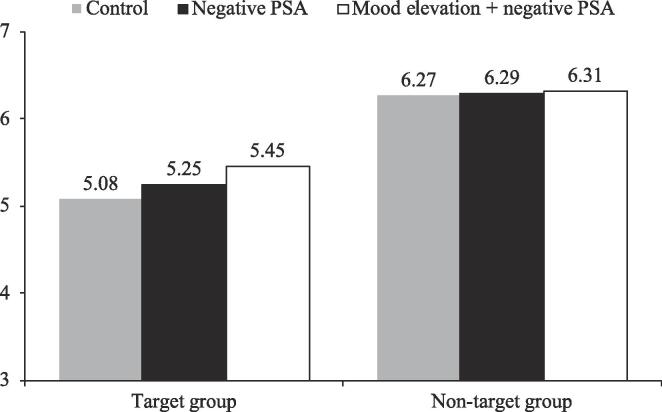
Moderating role of target group on attitude, Study 3.

**Fig. 8:**
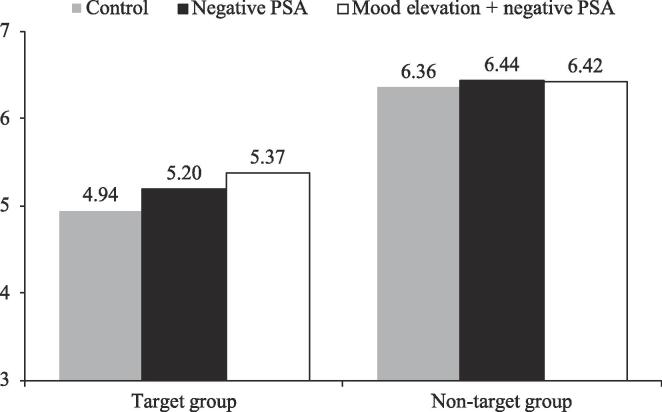
Moderating role of target group on behavioral tendency, Study 3.

Subsequent pairwise comparison analyses showed that, for attitude (Fig. 7), mood elevation significantly increased the attitude relative to the negative PSA condition for the target group (5.45 vs. 5.25; *SE* = 0.10, *p* < .05). Mood elevation + negative PSA also increased the attitude relative to the control condition for the target group (5.45 vs. 5.08; *SE* = 0.11, *p* < .001). Similarly, for behavioral tendency (Fig. 8), mood elevation increased the behavioral tendency relative to the negative PSA condition for the target group (5.37 vs. 5.20; *SE* = 0.11, *p* < .1). Mood elevation + negative PSA also increased behavioral tendency relative to the control condition for the target group (5.37 vs. 4.94; *SE* = 0.11, *p* < .001). Respondents in the negative PSA condition also had a higher behavioral tendency than those in the control condition for the target group (5.20 vs. 4.94; *SE* = 0.11, *p* < .05). These results suggested that mood elevation was more effective in improving the effectiveness (in terms of attitude and behavioral tendency) of negative PSAs for the target group than for the non-target group.

*Discussion*. This study added to the findings of the first two studies in demonstrating the effect of mood elevation in improving the effectiveness of negative PSAs in the context of an important and contemporarily relevant issue. Moreover, we tested the moderating role of the target group (H3) and found that mood elevation was more effective in enhancing message acceptance of the negative PSAs for those consuming wild animals frequently than for those who did not. Further, the results from analyzing the full sample and another reduced sample with a different sampling method also replicated these findings (see Web Appendix).

In this study, the mood elevation manipulation was adopted from real ads on TV and social media. We believe similar manipulations can be applied with negative PSAs in practice. Negative PSAs can thus communicate dire consequences in a way that yields improved message acceptance. Moreover, this study found that mood elevation was effective before negative information was shown in a PSA, thus suggesting that embedding negative PSAs before (studies 1 and 2) or after (study 3) mood-elevating programs in practice could both enhance their effectiveness.

In this study, the negative PSA did not decrease the message acceptance relative to the control condition. Although we did not predict the effect of the negative PSA conditions relative to the control condition, it may be helpful to understand the reason for this finding. We believe this result occurred because the negative PSA used in this study was designed to be informative and helpful, not merely negative and threatening. Further research should examine the interaction between the level of threat and usefulness of negative PSAs on message acceptance. In this study, we also tested the mediating role of mood through a moderated mediation model (model 8 in PROCESS; target group as a moderator), but the model was not significant. We speculate that this is due to the sequence of the manipulations in this study (the mood-elevating elements preceded the negative PSA instead of succeeded it). Future research could further investigate the mechanism and its relationship with the sequence of mood elevation manipulation.

## 6 General discussion

Negative PSAs often play an important role in communicating important information to the public and in increasing public awareness. Despite these benefits, sometimes they fail to achieve their desired impact due to viewers’ defensive reactions to negative and threatening information. This is especially likely when the target recipients of the negative PSA are those currently engaging in the misbehaviors noted in the PSA. In this research, we proposed and tested two solutions to improve the effectiveness of negative PSAs: mood elevation and self-affirmation. We found that mood elevation through incidental mood manipulations (study 1) and mood-elevating elements embedded into real PSAs (study 2 and study 3) are effective in increasing message acceptance of negative PSAs. Also, mood elevation can be effective both when it occurs before (study 1 and study 2) and after (study 3) the negative information in the PSA is shown. Additionally, we observed that self-affirmation can increase message acceptance through mood elevation, even when it fails to reduce identity threat. In other words, practitioners can use mood-elevating self-affirmation to enhance recipients’ acceptance of negative PSAs even when they cannot identify the relevant and important identity domains of their recipients.

This research contributes to the literature on persuasion by demonstrating two solutions to improve the effectiveness of negative PSAs. Through three studies with large consumer samples, we consistently showed the effectiveness of mood elevation (through direct manipulation or self-affirmation) in increasing acceptance of negative PSAs. We also demonstrated several successful implementations of these strategies. These implementations offer useful insights to PSA practitioners when designing their messages. For example, as in study 2, marketers can embed mood-elevating elements into PSAs. Alternatively, as in study 3, they can also embed the negative PSAs in the context of mood-elevating programs, such as before or after certain TV programs, advertisements, or comedy programs. Marketers can also consider inserting negative PSAs in the context of affirming messages, such as before or after TV shows that affirm family values. These strategies should help marketers convey the necessary negative information and avoid unintended outcomes, such as defensiveness.

This research also contributes to the literature on self-affirmation by providing another route other than identity threat reduction through which self-affirmation could operate in reducing defensiveness. Past work has debated whether the self-affirmation effect could be explained by mood. Some scholars (e.g., Koole et al., 1999, Raghunathan and Trope, 2002) have suggested that an elevated mood may explain the effects of self-affirmation. Others(e.g., Sherman & Cohen, 2006) have provided evidence against this mood explanation—for example, by showing that self-affirmation does not necessarily influence mood (e.g., Fein & Spencer, 1997). Our findings suggest that mood elevation and identity reduction may be two independent routes for self-affirmation to reduce defensiveness. The results show that, even when self-affirmation does not reduce identity threat, it can also augment message acceptance. In particular, our findings suggest that self-affirmation can increase message acceptance through enhancing mood. These findings thus provide insight to past debates regarding the role of self-affirmation and mood elevation in message acceptance. On a broader level, our findings suggest that identifying the conditions when self-affirmation is more likely to function through elevating mood and when it is more likely to function through threat reduction is an important future direction for self-regulatory research. Also, despite our having identified practical and effective solutions to improve the acceptance of negative PSAs in this research, we believe that the underlying reasons for these effects still need further elucidation. In our studies, we found significant direct effects of mood elevation and self-affirmation on message acceptance in addition to the indirect effect of mood (study 1), and the mediating effect of mood was not found when the sequence of the negative PSA and mood elevation was reversed (study 3). These results could reflect noisy measures, but they might also imply that there exist other factors (in addition to mood) driving the positive effects of mood elevation and self-affirmation. For example, mood elevation or self-affirmation may reduce recipients’ negative rumination on the negative and threatening information in the negative PSAs (e.g., Koole et al., 1999), which could reduce respondents’ cognitive load and therefore enhance their processing of the negative information (e.g., Rustagi & Shrum, 2018). Future research could further investigate such possibilities.

## Declaration of Competing Interest

The authors declare that they have no known competing financial interests or personal relationships that could have appeared to influence the work reported in this paper.

## Figures and Tables

**Figure:**
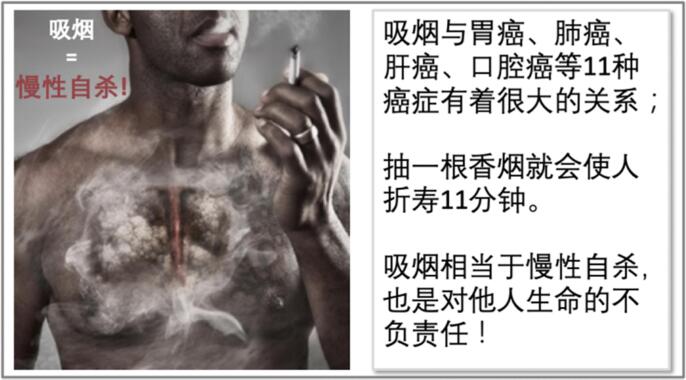


**Figure:**
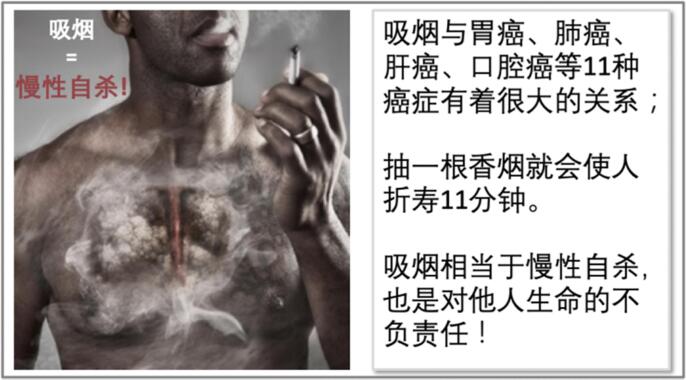


**Figure:**
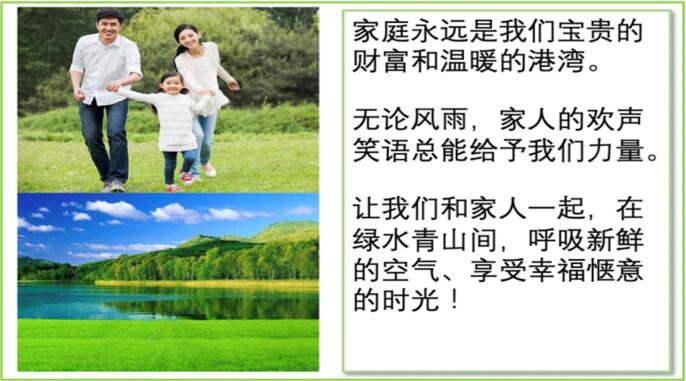


**Figure:**
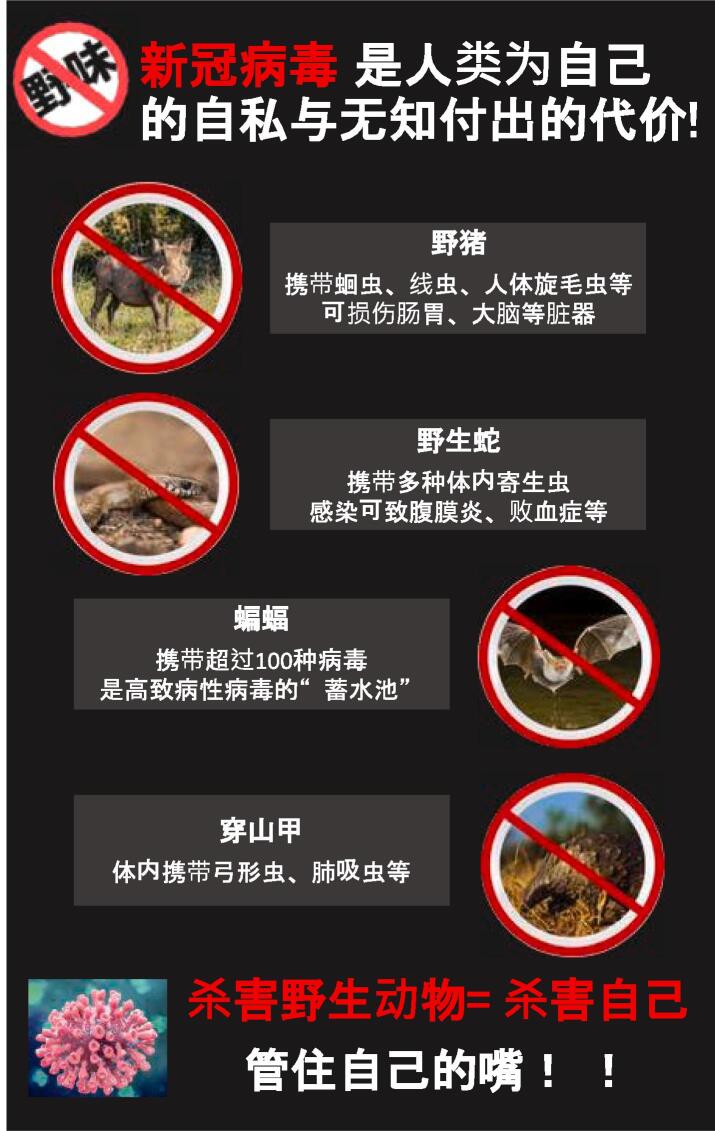


**Figure:**
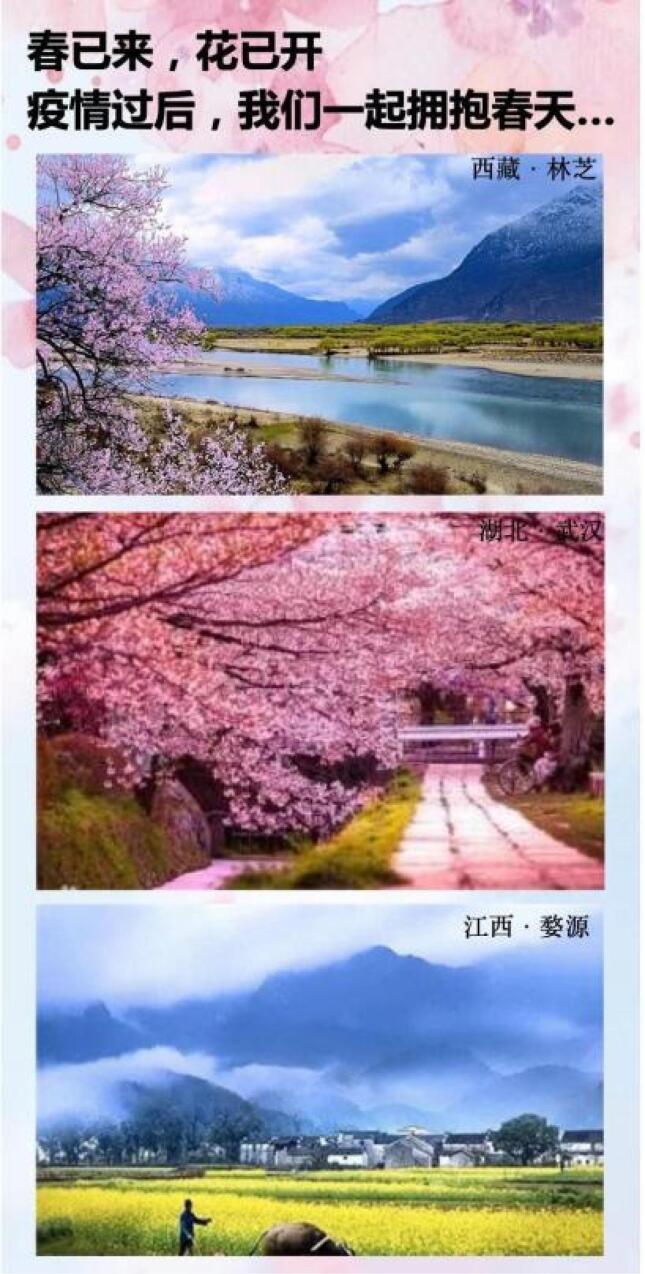


## Appendix A Negative PSA manipulations in study 1

1. Have you ever downloaded pirated software?
2. Have you ever downloaded pirated music?
3. Have you ever downloaded pirated games?
4. Have you ever downloaded pirated TV shows?
5. Have you ever downloaded pirated movies?
6. Have you ever downloaded pirated books?
7. Have you ever downloaded pirated pdfs?

According to our prior research on piracy, many individuals believe that pirating is like stealing. It is unethical and irresponsible behavior that could lead to negative impacts on society. Do you agree with them? (You do not need to answer this question right now).

## Appendix B Stimuli in study 2

### Negative PSA condition:

(Translation of the negative PSA)

Smoking = Slowly killing yourself!

Smoking has a great relationship with 11 types of cancers, including gastric cancer, lung cancer, liver cancer, and oral cancer.

Smoking a cigarette costs you 11 min of life.

Smoking is slowly killing yourself and is also irresponsible for the lives of others!

## Negative PSA + mood elevation condition:

(Translation of the positive PSA)

Family is always our precious wealth and warm harbor.

No matter what happens, the smile of our families can always cheer us up.

Let’s breathe fresh air and enjoy a happy time in the beautiful nature with our families!

## Appendix C Stimuli in study 3

### Negative PSA:

(Translation of the negative PSA)

The Coronavirus is the price that human beings pay for their selfishness and ignorance!

Wild boar: Carrying roundworms, nematodes, and human trichinellas that can damage organs, such as intestines, stomach, and brain.

Wild snake: Carrying a variety of internal parasites that can cause peritonitis or sepsis if infected.

Bat: Carrying more than 100 viruses, it is a reservoir of highly pathogenic viruses.

Pangolin: Carrying toxoplasma and lung flukes.

Kill wild animals = kill yourself!

Control your mouth!!

## Mood elevation manipulation:

(Translation)

Spring is coming and the flowers are blossoming.

After the Corona epidemic, let us embrace the spring together.

Picture 1: Linzhi, Tibet; Picture 2: Wuhan, Hubei; Picture 3: Wuyuan, Jiangxi

Although the pandemic is almost under control, we still need to remember the following lessons.
