# Supplementary material for: The route to improve the effectiveness of negative PSAs
Source: J Bus Res. 2021 Feb;123:669–82. doi: 10.1016/j.jbusres.2020.10.028 (PMC7772803; doi:10.1016/j.jbusres.2020.10.028)
Supplement: Supplementary data 1 [file mmc1.pdf]

Web Appendix

Supplemental Analyses in Study 3

1. Full sample results

We had 12,342 respondents (48.7% female; M_{age} = 37.85, SD = 14.40) in total. Since nearly 82% of them had never eaten any wild animals in the past, and 94.54% indicated a frequency of once a year or less, the sample size was severely unbalanced between the two target groups. Therefore, we only tested the main effect of mood elevation on message acceptance of the negative PSA with the full sample.

Message Acceptance. We conducted a MANCOVA with the three PSA conditions as an independent variable, the three indices (attitude, behavioral tendency, voting behavior) as dependent variables, and age, gender, education, province (from Hubei Province or not), living area (urban or rural area), number of children, family income, and frequency of eating wild animals as covariates. The results showed that the effects of the control variables were all significant (p's $< .05$). More importantly, after controlling for these variables, the effect of PSA condition was still significant ($F = 16.96$, Wilks' Lambda = .99; $p < .001$), thus suggesting significant differences among the three PSA conditions. Further, the results revealed that the three PSA conditions differed significantly on all three dependent variables (attitude: $F(2, 12330) = 39.17$, $p < .001$; behavioral tendency: $F(2, 12330) = 34.36$, $p < .001$; voting behavior: $F(2, 12330) = 4.53$, $p = .01$). Following pairwise analysis showed that, for attitude (Figure 1a), relative to respondents in the negative PSA condition, respondents in the mood elevation + negative PSA condition reported a significantly more favorable attitude toward the anti-wild-animal-consumption statements (6.13 vs. 6.20; $SE = .02$, $p < .001$). Further, both PSA conditions

had a significantly more favorable attitude than those in the control condition (6.01; *SE's* = .02, *p's* < .001).

For behavioral tendency (Figure 1b), the results were similar to those on the attitude measure: Relative to respondents in the negative PSA condition, those in the mood elevation + negative PSA condition reported a higher behavioral tendency to stop (their or others') wild animal consumption in the future (6.27 vs. 6.30; *SE* = .02, *p* = .09). Furthermore, both PSA conditions induced a higher behavioral tendency than in the control condition (6.14; *SE's* = .02, *p's* < .001).

For voting behavior (Figure 1c), relative to respondents in the negative PSA condition, those in the mood elevation + negative PSA condition were more likely to support the ban on the consumption and sale of wild animals (2.90 vs. 2.99; *SE* = .04, *p* < .05). Furthermore, relative to respondents in the control condition, those in the mood elevation + negative PSA condition were more likely to support relevant laws (2.88 vs. 2.99; *SE* = .04, *p* < .01). However, the respondents in the negative PSA condition were not significantly different from those in the control condition in the likelihood of supporting these laws (2.90 vs. 2.88; *SE* = .01, *p* > .1). These results show that mood elevation can increase the effectiveness of negative PSAs in terms of attitude, behavioral tendency, and voting behavior.

Figure 1a (Web Appendix) here.

Figure 1b (Web Appendix) here.

Figure 1c (Web Appendix) here.

2. Stratified random sampling 2.0

To test the robustness of the moderation effect, we drew another subsample using a different stratified sampling method. Specifically, we retained all respondents eating wild animals in the past as the target group (N = 2,251; 18.24% of all respondents) and then randomly drew the same number of respondents from the remaining sample members as the non-target group. The sampling was based on the sample size in each PSA condition of the target group (N = 779, 759, 713 in the control, negative PSA, and mood elevation + negative PSA condition, respectively) such that there were equal groups of respondents who did and did not eat wild animals and in each PSA condition. We then conducted analyses using these 4502 respondents (46% female; M_{age} = 37.95, SD = 14.25).

Moderation Effect. We conducted a two-way MANCOVA with the PSA condition and the target group as independent variables, the three indices as dependent variables, and age, gender, education, province (from Hubei Province or not), living area (urban or rural), number of children, family income, and frequency of eating wild animals as covariates. Results showed that the effects of the control variables were all significant (p's $< .01$) except for living area ($p > .1$). Furthermore, after controlling for these variables, the main effect of the PSA condition ($F = 8.32$, Wilks' Lambda = .99; $p < .001$) and target group ($F = 274.90$, Wilks' Lambda = .85; $p < .001$) were still significant. More importantly, the interaction between the PSA condition and target group was significant ($F = 2.72$, Wilks' Lambda = 1.00; $p = .01$), suggesting that the effect of PSA condition on the dependent variables differed between the target and non-target groups. Moreover, we found that the interaction was significant for attitude ($F(2, 4488) = 4.27$, $p < .05$) and behavioral tendency ($F(2, 4488) = 4.77$, $p < .01$) but non-significant for voting behavior ($F(2, 4488) = 2.18$, $p = .11$). Specifically, contrast analysis showed that the effect of the PSA condition on attitude was stronger for the target group ($F(2, 4488) = 22.42$, $p < .001$) than for the

non-target group ($F(2, 4488) = 3.35$, $p = .04$); the effect of the PSA condition on behavioral tendency was also stronger for the target group ($F(2, 4488) = 17.99$, $p < .001$) than for the non-target group ($F(2, 4488) = 2.84$, $p = .06$). Then, a pairwise comparison analysis showed that, for attitude, mood elevation increased the attitude significantly relative to the negative PSA condition for the target group (5.80 vs. 5.68; $SE = .06$, $p < .05$) but not for the non-target group (6.14 vs. 6.11; $SE = .06$, $p = .56$). In addition, both PSA conditions had induced a significantly more favorable attitude than the control condition for the target (5.44; *p's* < .001) and non-target groups (6.00; mood elevation + negative PSA condition vs. control condition; $p = .01$; negative PSA condition vs. control condition: $p =.06$). For behavioral tendency, mood elevation did not increase respondents' behavioral tendency significantly relative to the negative PSA condition for the target group (5.80 vs. 5.77; $SE = .06$, $p = .58$) nor for the non-target group PSA conditions (6.24 vs. 6.15; $SE = .06$, $p = .10$). Respondents in the mood elevation + negative PSA condition reported a higher behavioral tendency than those in the control condition, both for the target (5.80 vs. 5.50; $SE = .06$, $p < .001$) and non-target groups (6.24 vs. 6.11; $SE = .06$, $p < .05$). Results also revealed that the negative PSA increased the behavioral tendency relative to the control condition for the target group (5.77 vs. 5.50, $SE = .06$, $p <.001$) but not for the non-target group (6.15 vs. 6.11; $SE = .06$, $p = .48$).
