# Supplementary material for: The route to improve the effectiveness of negative PSAs
Source: J Bus Res. 2021 Feb;123:669–82. doi: 10.1016/j.jbusres.2020.10.028 (PMC7772803; doi:10.1016/j.jbusres.2020.10.028)

Web Appendix Figures

Figure 1a (Web Appendix). Attitude, Study 3 full sample.

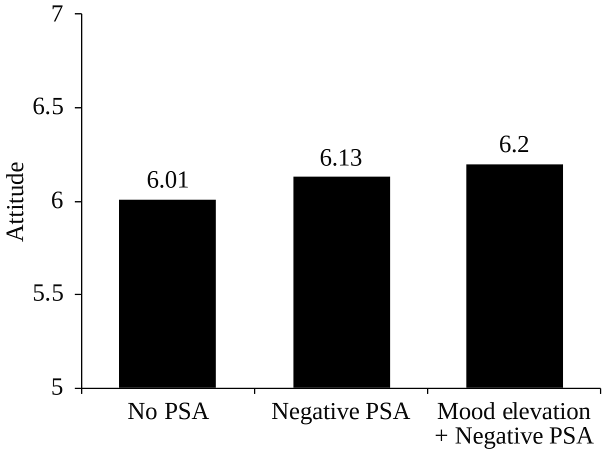

Figure 1b (Web Appendix). Behavioral tendency, Study 3 full sample.

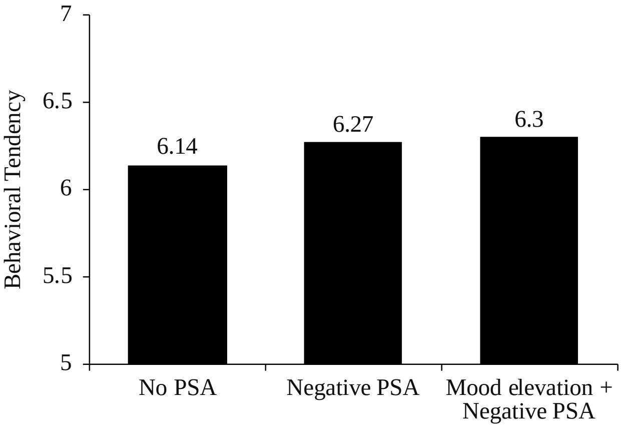

Figure 1c (Web Appendix). Voting behavior, Study 3 full sample.

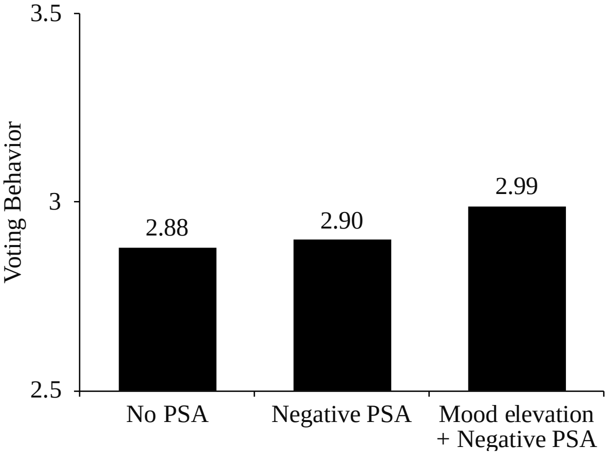

Supplement: Supplementary data 2 [file mmc2.pdf]
